# Olfactory mucosa tissue-derived mesenchymal stem cells lysate ameliorates LPS-induced acute liver injury in mice

**DOI:** 10.1186/s12890-022-02204-7

**Published:** 2022-11-11

**Authors:** Zhe Wang, XingXing Zhang, Liuyao Qi, Wenjing Feng, Yahan Gu, Yuting Ding

**Affiliations:** 1 School of Medicine, Jiangsu University, Zhenjiang, 212013 P. R. China; 2 Department of Rehabilitation, Changshu No. 2 People’s Hospital, Changshu Hospital affiliated the Xuzhou Medical University, No.68, Haiyunan Road, Changshu, Jiangsu China

**Keywords:** OM-MSCs lysate, LPS, ALI, Anti-inflammation, Recovery

## Abstract

**Background:**

Acute liver injury (ALI) induced by sepsis seriously endangers the health of human beings every year. Mesenchymal stem cells (MSCs) lysate containing various regulators had a positive effect on anti-inflammation, hoping to provide a promising strategy in ALI.

**Methods:**

Olfactory mucosa-derived mesenchymal stem cells (OM-MSCs) were extracted and identified. The collected OM-MSCs were prepared after repeated freeze–thaw in phosphate buffer solution (PBS). Then, OM-MSCs lysate was filtered for future experiments. To understand the composes of OM-MSCs clearly, we detected the components of OM-MSCs lysate by western blotting. *In vitro*, OM-MSCs lysate was applied to evaluate the effects on normal human liver cells (LO-2) under stimulation of LPS. Lipopolysaccharide (LPS) was also injected intraperitoneally to build ALI model in mice. We further assessed the anti-inflammatory capacity of OM-MSCs lysate on ALI *in vivo *by aminotransferase determination, pathology observation, and immunohistochemical staining. Moreover, the immunoblot technique was performed to recognize the changes in inflammatory factors and related proteins.

**Results:**

In this study, we found that OM-MSCs lysate could protect structure effectively, improve the plasma aminotransferases, diminish inflammation by releasing interleukin-10 (IL-10) and transforming growth factor-beta (TGF-β). A significant decrease in tumor necrosis factor-α (TNF-α) also occurred under the treatment of OM-MSCs lysate. In addition, trophic factors originating from OM-MSCs lysate provided a supportive micro-environment for liver recovery. Especially, up-expression of vascular endothelial growth factor (VEGF) *in vivo* revealed that OM-MSCs might have a great potential for healing.

**Conclusions:**

Our results demonstrated that OM-MSCs lysate could alleviate LPS-induced ALI via decreasing inflammatory cytokines and promoting recovery.

**Supplementary Information:**

The online version contains supplementary material available at 10.1186/s12890-022-02204-7.

## Introduction

Sepsis is an excessive systemic inflammation accompanied by redness, swelling, fever, pain, and loss of function [1]. The overwhelming release of pro-inflammatory cytokines eventually leads to organ dysfunction [1, 2]. As a regulator of the inflammatory process and a target of host response, liver injury is strongly associated with lower survival in patients with sepsis [3, 4]. However, there is still no specific therapy and comprehensive understanding of ALI in sepsis.

Unlike other MSCs, OM-MSCs exhibited more excellent anti-inflammatory abilities [5, 6]. Easy sampling and less trauma also provide new hope for clinical application [7]. Although OM-MSCs own great potential in transplantation, the problems of low survival rate and poor differentiative capacity caused by the innate immune response, oxidative stress, and ischemic microenvironment still trouble us [8–11]. Recently, some studies attributed MSCs’ potent therapeutic function to paracrine effects [12–14]. Secretome from MSCs ameliorated cell proliferation, activation, and migration, eventually improving the microenvironment [15–18]. A novel cell-free therapy has been generating tremendous research interest in research [19, 20]. As a molecular factory, MSCs themselves have more prosperous factors inhibiting inflammation and reducing apoptotic than secretome [21, 22]. Notably, the paper already reported that MSCs lysate could alleviate inflammatory disorders [23]. Given that, we sought to investigate the effects of soluble molecules in OM-MSCs lysate on ALI induced by LPS and clarify the potential mechanism.

## Materials and methods

### Isolation and identification of OM-MSCs

As a previous study reported [24], OM-MSCs were isolated from olfactory mucosa in C57 purchased from Nanjing Qing long shan company. The snipped nasal mucosa tissue was cultured in Dulbecco’s modified Eagle medium/HAM’S F12 (DMEM/F12) containing 15% fetal bovine serum (FBS) purchased from Gibco., Ltd (US) for three days. OM-MSCs were passaged and expanded until the degree of integration achieved 80%. To identify the surface marks, the purified OM-MSCs were detected by immunofluorescence of Nestin, Vimentin, and S100.

### Preparation and analysis of OM-MSCs lysate

To prepare OM-MSCs lysate, we adopted a modified procedure according to the previous study [23]. In brief, when OM-MSCs in good condition at passage 3–5 reached 80% confluency, they were collected using trypsin–EDTA after washing with PBS twice. Then, the obtained OM-MSCs were counted and impregnated with PBS to make concentration achieved at 1.5 × 10^6^/300 μL according to reference [22]. Following that, cell suspension underwent a freeze–thaw cycle four times and were filtered by a 0.22 μm membrane filter. The OM-MSCs lysate was prepared for experiments *in vitro* and *vivo*, and also been analyzed by western blotting. IL-10 is a vital immunoregulatory cytokine that exert essential functions to maintain homeostasis through restriction of excessive inflammation, upregulation of innate immunity, and promoting tissue repairing [25]. As a crucial enforcer of immune homeostasis and tolerance, TGF-β is also central to immune suppression [26]. Sonic hedgehog (SHH), Collagen II, and Laminin play an important role in the construction of a regenerative environment [27–29]. It makes more sense to evaluate the potential of OM-MSCs lysate via testing these cytokines. Consequently, we added loading buffer into OM-MSCs lysate obtained from the above method to make samples, and loaded them into polyacrylamide gel electrophoresis (SDS-PAGE) system to test IL-10, TGF-β, SHH, Collagen II, and Laminin. 

### Assessment of OM-MSCs lysate *in vitro*

LO-2 purchased from Fenghuishengwu Co.Ltd. was cultured in 1640 RAMI medium containing 10% FBS. The LO-2 was stimulated by 10 ng/mL LPS to build cell models for two days. To confirm whether OM-MSCs lysate could rescue LO-2 against inflammatory injury, 100 μL lysate from 1 × 10^6^ OM-MSCs was immediately added into a culture dish where the LO-2 counting number is 6 × 10^6^ for 12 h after the establishment of the cell model. Then, the LO-2 under the intervention of OM-MSCs lysate was collected. Western blotting was used for the expression of IL-10, myeloperoxidase (MPO), and TNF-α *in vitro* and the bands were cut according to molecular weight prior to hybridization with antibodies. Furthermore, immunofluorescence was performed to confirm the anti-inflammatory properties of OM-MSCs lysate and to explore whether OM-MSCs lysate could promote proliferation via assessment of KI-67.

### Animal models

Male C57BL/6 mice (20–25 g) aged 6–8 weeks were purchased from Nanjing Qing long shan company. Mice were kept at 23 ± 2℃ and humidity at 45–55%. All experimental protocols on animals were approved by the regional committee for the study of animals and followed guidelines for the correct use of animals in research. Thirty mice were divided into three groups: control group, LPS group, OM-MSC lysate group. All ALI model mice were induced by intraperitoneal injection of LPS (5 mg/kg). Each mouse from the intervention group was given 300 μL OM-MSCs lysate [22]. Finally, mice were sacrificed after 6 h of LPS stimulation.

### Detection of Serum ALT and AST

Blood was collected from the heart with a 1 mL needle and kept at room temperature for one hour. Then serum was separated by centrifugation at 10000 rpm for 5 min at 4℃. After confirming no hemolysis in serum, the serum alanine aminotransferase (ALT) and aspartate aminotransferase (AST) levels were ascertained to evaluate liver function using ALT/GPT tests and AST/GOT tests (Nanjing Jiancheng Bioengineering institute). In short, 100 μL serum was added and incubated according to the manufacturer of the kit. The absorbance at 450 nm was analyzed to assess the damage.

### Liver histological analysis

The liver tissue samples were fixed with 4% paraformaldehyde and then embedded in paraffin. The 5 μ thick sections were stained with hematoxylin and eosin (H&E). All of the images were captured by microscope. The extent of histological changes was scored according to the previous study [30].

### Periodic acid Schiff staining and immunohistochemical analysis

Liver tissue was deparaffinized and rehydrated in xylene and graded alcohols. The sections were blocked at room temperature for 30 min and incubated overnight at 4 °C with anti-IL-10, anti-TNF, and anti-VEGF antibodies (BOSTER Biological Technology co.ltd). Subsequently, the sections were rinsed, and incubated at 37 °C for 30 min with secondary antibodies. Ultimately, the DAB kit was used to color and followed by counterstaining with hematoxylin. What’s more, periodic acid-schiff staining (PAS) was applied to assess a variety of processes in the liver [31].

### Western-blotting analysis

The frozen liver tissues (*n* = 3 per group) were ground and homogenized, then extracted with RIPA solution containing protease inhibitor cocktail on ice for 30 min. The protein samples were mixed with 5 × loading buffer and boiled for 10 min. 10 μL processed samples were added in a 10% (w/v) gel, separated by SDS-PAGE, and transferred onto polyvinylidene fluoride (PVDF) membrane. The membrane was blocked by 5% nonfat dry milk and incubated with primary antibodies including IL-10, TNF-α, TJP, and Actin overnight at 4 °C. Prior to hybridization with antibodies, blots were cut according to molecular size. Then, the target bands were visualized and analyzed by Image J software after the combination of secondary IgG-Horseradish Peroxidase (HRP)-conjugated antibodies.

### Statistical analysis

All data were expressed as the means ± standard of three independent experiments. Statistical significance of the differences was analyzed by one-way analysis of variance (ANOVA) using the SPSS statistical 23.0 software (SPSS Inc., Chicago, IL, USA). *p* < 0.05 meant a statistically significant level.

## Results

### Culture and identification of surface markers of OM-MSCs

**Fig. 1:**
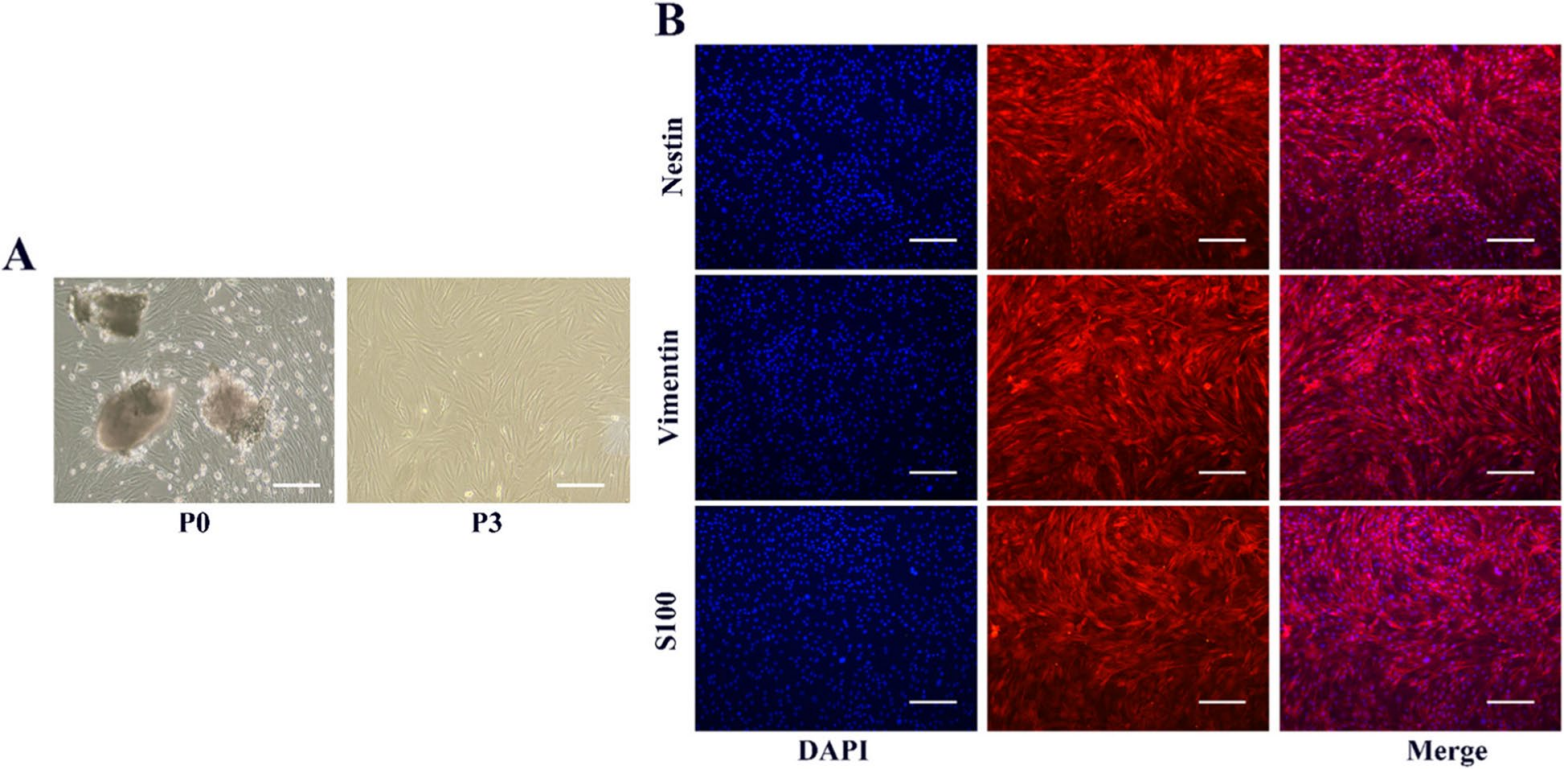
Extraction and identification of OM-MSCs. **A** cell morphology in different stages. **B** Identification of OM-MSCs through surface markers like Nestin, Vimentin, and S100. 200 × magnification, bar = 100 μm

Under the technique of explanted tissue culture, OM-MSCs migrated out from the nasal mucosa. After three days, typical spindle-like shaped cells adhered to the surface and spread out with a high proliferation rate (Fig. 1A). Furthermore, OM-MSCs typically expressed some essential antigenic markers, which proved the cell stemness (Fig. 1B). The results revealed that positive expression of Nestin, Vimentin, and S100 appeared on the cell surface [32], suggesting that high-purity OM-MSCs can be harvested simply and efficiently through subculture.

### Test of effective component in OM-MSCs lysate

**Fig. 2:**
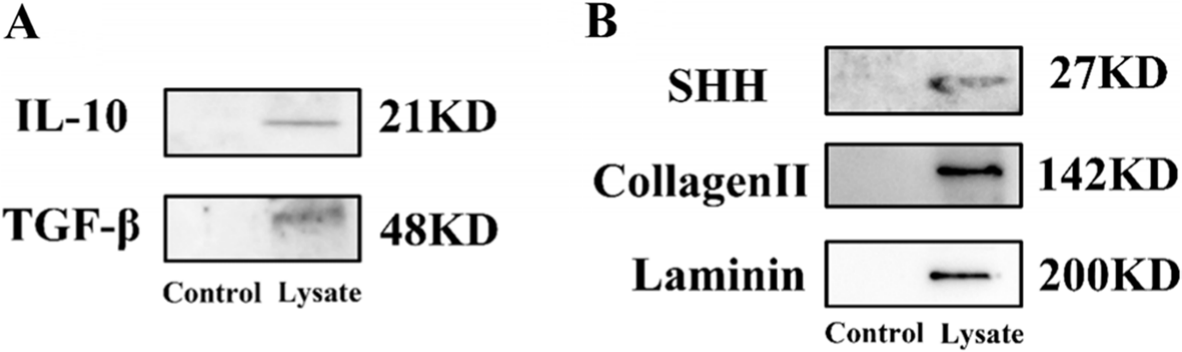
Components of OM-MSCs lysate. **A** Evaluation of anti-inflammatory potential through detection of IL-10 and TGF-β (**B**) Assessment of nutritional capacity via detection of SHH, Collagen II, and Laminin

To further explore whether OM-MSCs lysate contains beneficial factors, western blotting was applied. The results demonstrated the existence of IL-10 and TGF-β, indicating that OM-MSCs lysate owns a great potential in regulating the process of inflammation (Fig. 2A). Actually, the OM-MSCs lysate only exhibited partial ability. We further detected nutritional cytokines and promoting factors. Excitingly, the results didn’t disappoint us. SHH, accelerating the process of development in the embryonic period, was also proved. Likewise, Collagen II, and Laminin were distributed in OM-MSCs lysate (Fig. 2B). These proofs clarified the essential components of OM-MSCs lysate. All of the full-length blots/gels are presented in Figure S1.

### OM-MSCs lysate regulated inflammatory response

**Fig. 3:**
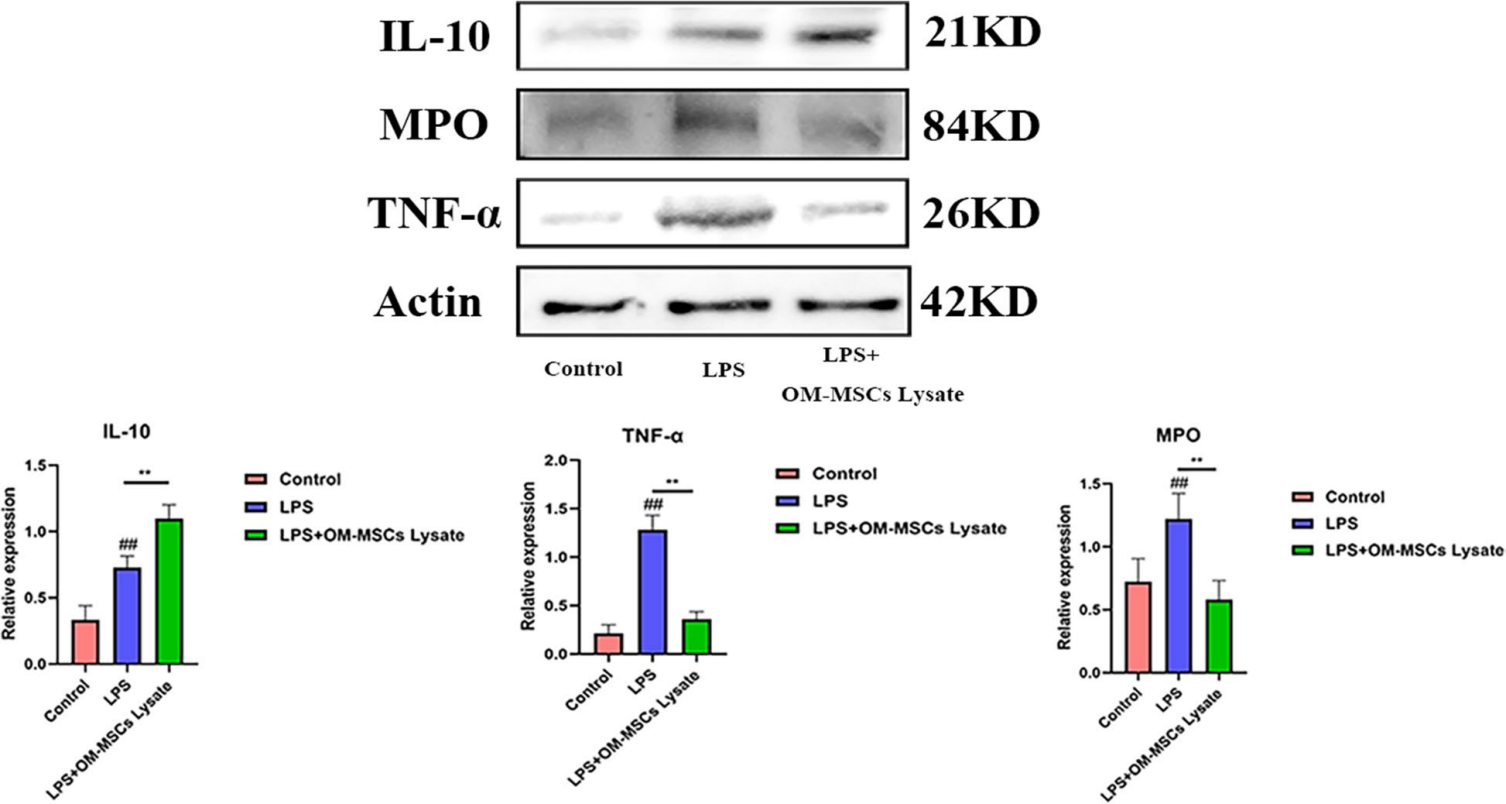
OM-MSCs lysate relieve LPS-induced liver inflammatory injuries. Compared to the LPS group, the OM-MSCs lysate group suppressed inflammation by detection of TNF-α and MPO via up-expression IL-10. Data are performed as the mean ± SD (*n* = 3). ##*p* < 0.05 *vs*. control alone, **p < 0.05

Though our data indicated the therapeutic potential of OM-MSCs lysate, the effect of OM-MSCs lysate is unclear. Consequently, we evaluated the changes in inflammation-related cytokines in LO-2. IL-10 is a great target in treating immune disease. Potent ability of anti-inflammation makes it become a research pot. The results showed that the level of IL-10 in LO-2 obtained a greater improvement in the LPS + OM-MSCs lysate group than LPS group (Fig. 3). To clearly explain the role of OM-MSCs lysate in LO-2, we deeply analyzed the changes of inflammatory cytokines. Interestingly, the data also revealed that the LO-2 exposed to OM-MSCs lysate own lower levels of MPO and TNF-α compared with the LPS group, which suggests that IL-10 may play an essential role during this process. The blots/gels are presented in Figure S2.

### Distribution and expression of inflammation-related factors and proliferative markers in LO-2

**Fig. 4:**
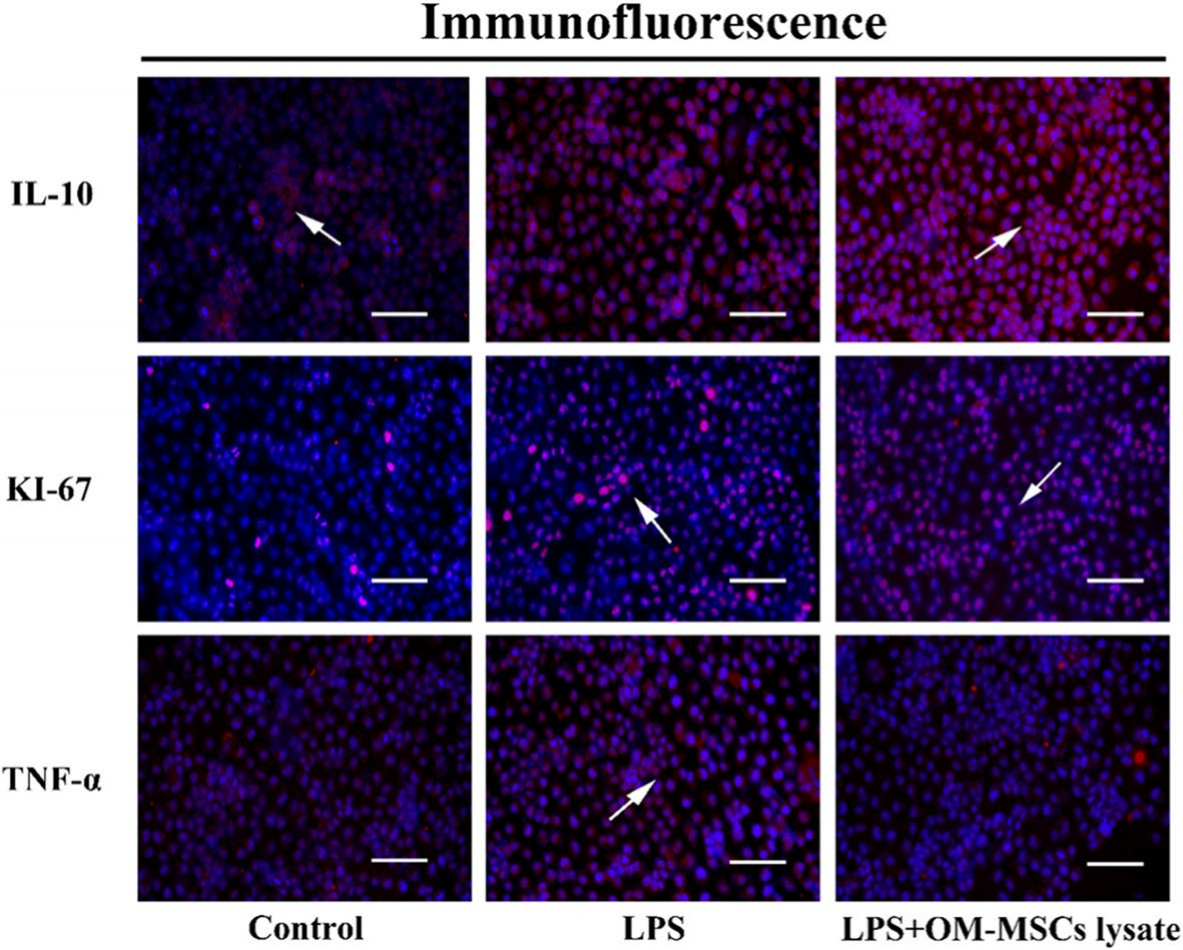
Evaluation of anti-inflammation and proliferative abilities of OM-MSCs lysate *in vitro*. The immunofluorescence analysis was performed with antibodies including IL-10, KI-67 and, TNF-α. 4′,6-diamidino-2-phenylindole (DAPI) was used to label the nuclei (blue). (bar = 100 nm)

Our results preliminarily showed that OM-MSCs lysate could suppress the process of inflammation via up-expression in LO-2. To confirm this phenomenon, we detected the distribution and expression of IL-10 and TNF-α with the support of immunofluorescence (Fig. 4). Consistent with the above data, massive IL-10 was distributed in LO-2 under the stimulation of OM-MSCs lysate. Meanwhile, only limited TNF-α was expressed in the OM-MSCs lysate group. Except for basial anti-inflammatory components in OM-MSCs lysate, the existence of nutritional factors is also considerable. For a better understanding of them, we evaluate the proliferative influence of OM-MSCs lysate on LO-2. As proliferative markers, the expression of KI-67 reflects the effects largely. The immunofluorescence results directly pointed out that OM-MSCs lysate could promote the growth of LO-2.

### OM-MSCs lysate inhibited the Release of Liver Enzymes

**Fig. 5:**
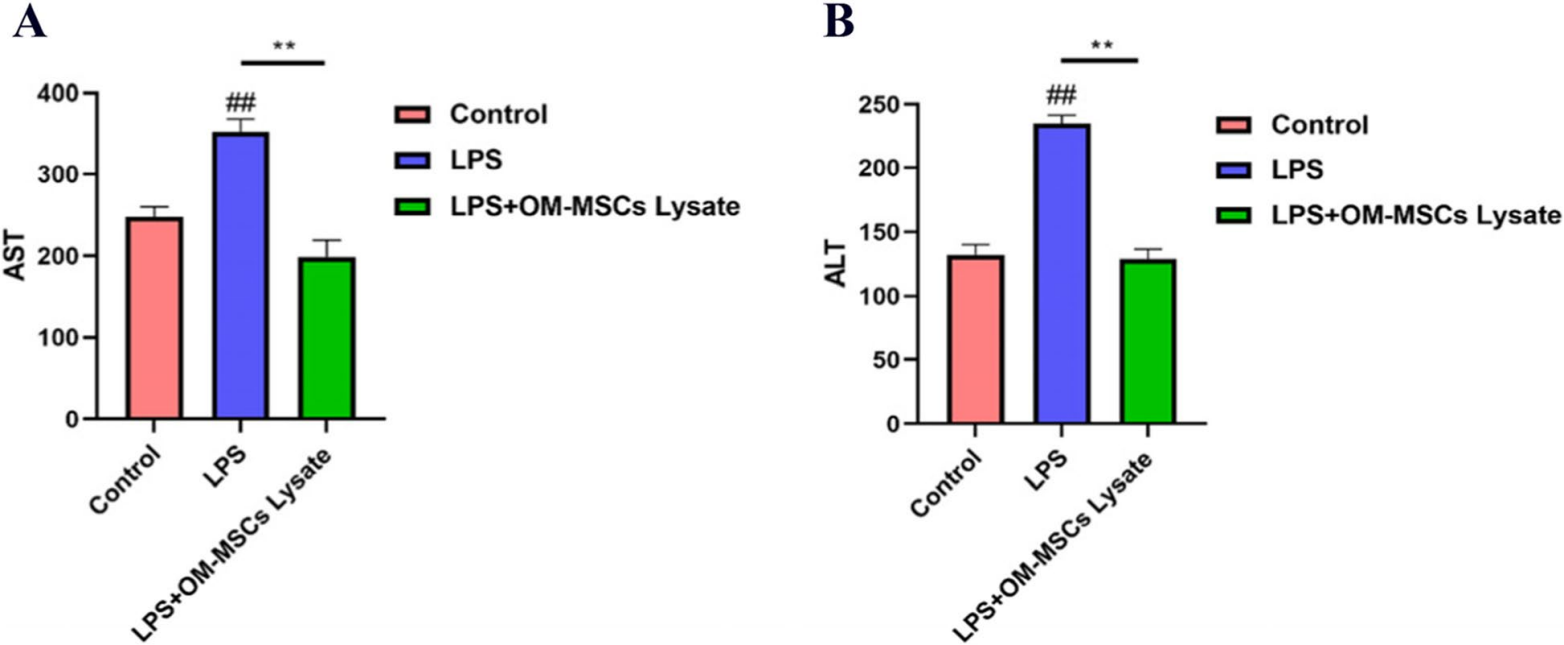
Serum levels of aspartate transaminase (AST) and alanine aminotransferase (ALT) in the experimental groups. **A** Detection of AST. **B** Test of ALT. All data were obtained from the serum of mice (*n* = 3 per group), and all data are mean ± SD (*n* = 3)

Variation of enzymes in the liver could reflect liver function to some extent. We found that LPS-induced ALI in mice obtained an obvious increase in AST and ALT (Fig. 5A-B), which indicates us the establishment of ALI achieved great success. What’s more, OM-MSCs lysate effectively decreased the degree of elevation of liver enzymes, reflecting the positive significance of OM-MSCs lysate in ALI. Our data showed that the level of AST/ALT in serum in the OM-MSCs lysate group significantly decreased by 80% compared with the LPS group (*p* < 0.05), which also indicated that it is of great research significance.

### Histological evaluation

**Fig. 6:**
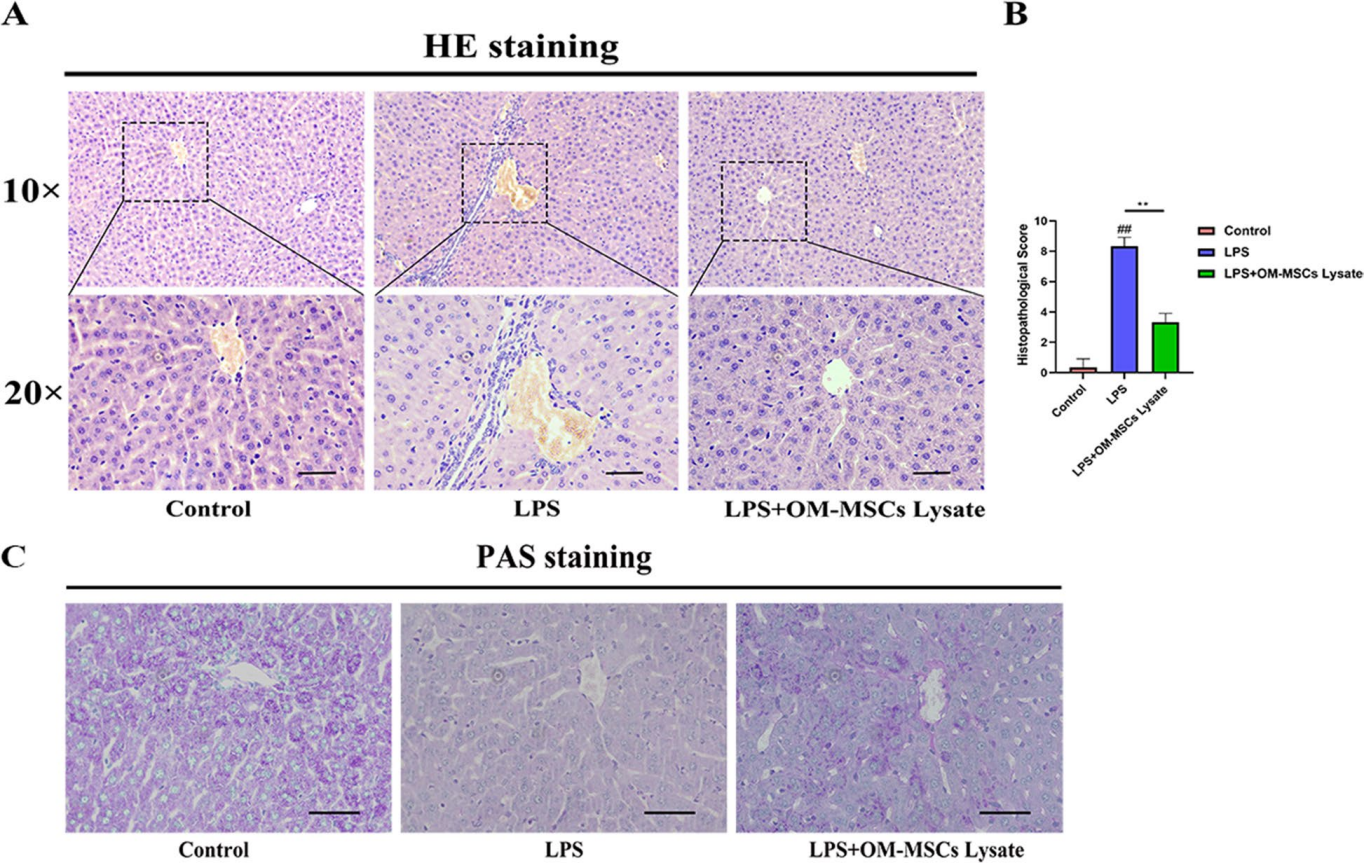
**A** H&E staining in the experimental groups. **B** HE scores. ##*p* < 0.05 vs. control alone, **p < 0.05. **C** PAS staining. (bar = 50 μm)

We next performed histochemical staining and investigated whether OM-MSCs lysate could improve the ALI in mice. Histological examination showed that extensive inflammatory cells infiltrated the vein in LPS group. Excitingly, our data showed that OM-MSCs lysate could alleviate this phenomenon effectively. The liver cells are arranged in an orderly fashion, with fewer inflammatory cells distributed in the OM-MSCs lysate group (Fig. 6A-B). Moreover, we evaluated glycogenesis alteration, which reflects the function of the liver. As vividly shown in the picture, the OM-MSCs group improved the function of liver glycogen synthesis significantly (Fig. 6C). Though LPS-induced inflammatory reaction seriously damaged the basial the function of the liver, OM-MSCs lysate group reserved unfavorable situation compared with the LPS group.

### OM-MSCs lysate suppressed the inflammatory cytokines and restored LPS-induced liver injury

**Fig.7:**
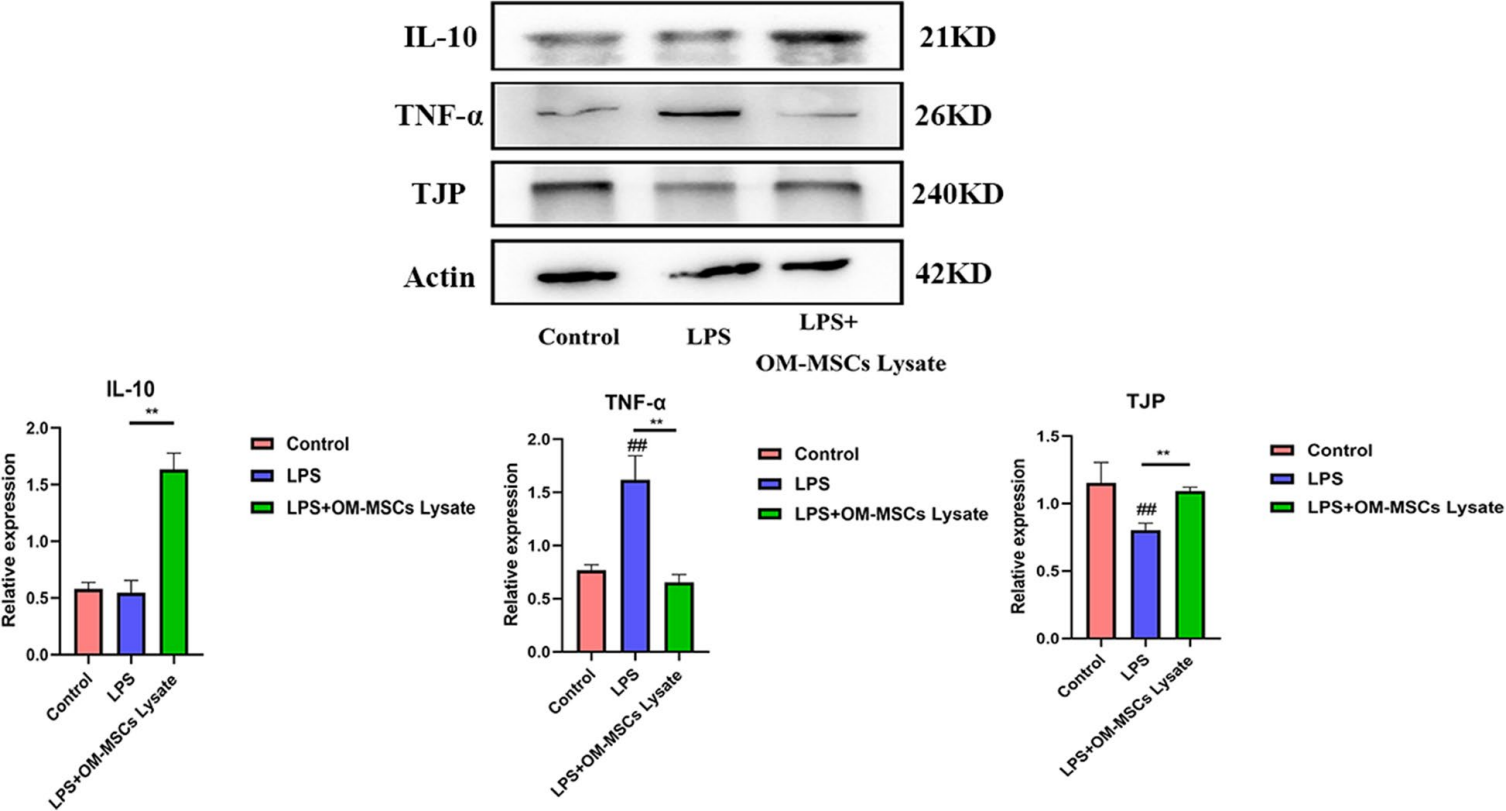
The expression of IL-10, TNF-α, and MPO in the experimental group. ##indicates the significant difference compared with the LPS group, *p* < 0.05, **p < 0.05

To verify the anti-inflammatory effects of OM-MSCs lysate in the liver, we detected the expression of inflammation-related proteins (Fig. 7). Consistent with results in vitro, IL-10 was expressed richly in the liver. Due to this finding, we deeply analyzed the expression of TNF-α. Obviously, the low expression of TNF-α hinted to us that IL-10 might play an indispensable role. Regulating the inflammatory response to inhibit inflammatory injury may be an effective treatment to improve LPS-induced ALI. Tight junction protein (TJP) is a tight link protein, and the protein content often reflects the degree of liver recovery. Consequently, we determined the level of TJP in the liver. It’s exactly what we thought the group under treatment of OM-MSCs lysate protects the liver effectively. The full-length blots/gels are presented in Figure S3.

### Immunohistochemical assessment

**Fig. 8:**
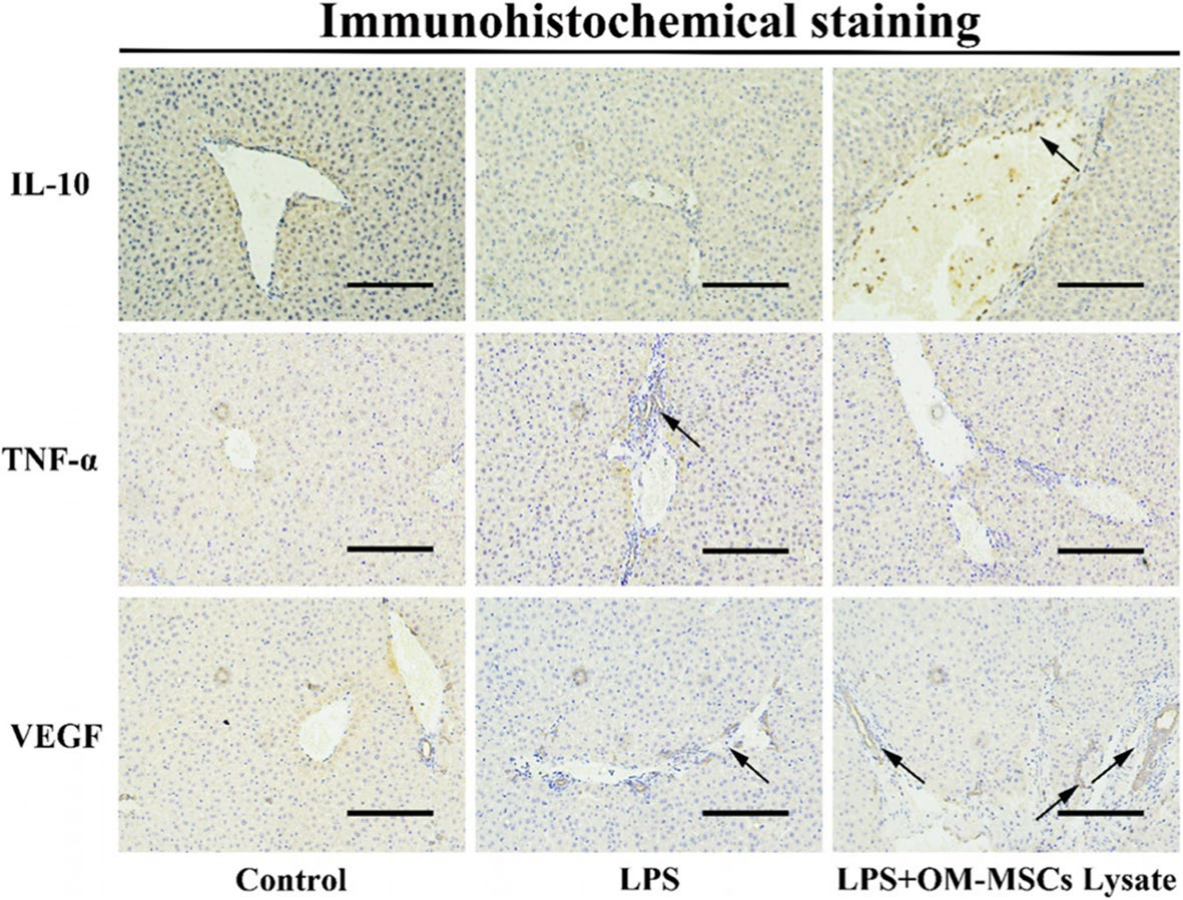
Effects of OM-MSCs lysate on sepsis-induced ALI in mice via detection of IL-10, TNF-α, and VEGF. The arrows indicate a strong positive expression. bar = 100 μm

To further confirm the effects of OM-MSCs lysate, we performed immunohistochemical staining. As we pointed out in Fig. 8, abundant IL-10 distributed around the vein in the OM-MSCs lysate group, and rich VEGF spread over the liver. Different from the LPS group, other positive effects also occurred in the OM-MSCs lysate group, including fewer soakage of inflammatory cells and low levels of TNF-α. In a word, we proved that OM-MSCs lysate prevents the destruction of inflammation via up-expression of IL-10 and promotes expression of angiogenesis via nutritional influence.

## Discussion

The results in this paper revealed that OM-MSCs lysate containing multifarious cytokines could alleviate the experimental ALI in mice via regulating inflammation. Through analyses of components in OM-MSCs lysate, we preliminarily proved essential cytokines existed in OM-MSCs lysate, indicating the anti-inflammatory potential of OM-MSCs lysate. Additionally, we found that OM-MSCs lysate significantly suppressed the level of proinflammatory cytokines *in vitro* and *vivo,* providing supportive evidence for the anti-inflammatory properties of OM-MSCs lysate. HE staining and immunochemical staining further disclosed the protective effects of OM-MSCs lysate. Taken together, the therapeutic influence of OM-MSCs lysate in LPS-induced ALI hopes to provide reference information for strategy.

MSCs are immunomodulatory and multipotent cells isolated from various tissues. Numerous studies have explored MSCs’ function in the inhibition of inflammation and tissue regeneration in different animal models in *vitro* [33–37]. However, due to the harsh living conditions for MSCs in lesions, the problem of the low survival rate of MSCs remained to be solved. Recently, MSCs cell-free therapy has become a new trend [38–40]. More and more papers attribute the effects of MSCs to the MSCs-derived secretome including growth factors, cytokines, anti-inflammatory mediators and exosomes [18]. Furthermore, it has been reported that MSCs lysate contains anti-inflammatory and regenerating factors and positively affects many experimental models [21, 22]. In addition, various surface molecules from the MSCs membrane mediate direct communication between cells. These surface molecules, like chemokine receptors, co-inhibitory molecules, cytokine receptors, and adhesion molecules endows MSCs lysate with potent immunosuppressive property to establish a tolerant microenvironment [41]. This evidence indicated that MSCs lysate could promote a favorable environment formation. Notably, researchers have already demonstrated that lysate of adipose tissue‑derived mesenchymal stem cells (ADSCs) could improve colitis. Moreover, the injection of a mixture of conditional medium and lysates from ADSCs also achieved great success in acute liver failure (ALI) [42]. However, awareness of the role of OM-MSCs lysate in hepatitis is still inadequate.

OM-MSCs are a particular type of mesenchymal stem cells derived from the nasal mucosa. Due to the special parts of tissue, easy and patient-friendly sampling becomes a bright point [7]. Same with other types of MSCs, the powerful restorative and anti-inflammatory abilities of OM-MSCs have been confirmed [43]. Transplantation of OM-MSCs with a functional scaffold also achieved promising progress [5, 44]. Whereas, OM-MSCs have not been further studied in cell-free therapy. As a key anti-inflammatory mediator, the cytokine called IL-10 is emerging as an attractive therapeutic target in human disease [45]. Abundant papers have proved anti-inflammatory response of IL-10 brings new hope for many immune diseases, such as autoimmune encephalomyelitis, crohn’s disease, ulcerative colitis and rheumatoid arthritis [46]. TGF-β, a master immune regulator, has also been well studied [47]. Excitingly, we found that both IL-10 and TGF-β existed in OM-MSCs lysate. The above results also revealed that the OM-MSCs lysate group could alleviate ALI via decreasing inflammatory factors. A new therapy based on OM-MSCs lysate in treating sepsis-induced ALI has a promising protentional to become the well-being for patients.

Though OM-MSCs lysate had a satisfactory effect on experimental models, our study still has some limitations. In this paper, we reported that OM-MSCs lysate had a comprehensive efficacy. However, because of the existence of various bioactive compounds, the main component of the composite is still unclear. We just uncovered that OM-MSCs lysate own main favorable factors and proved that OM-MSCs lysate could mediate the immunoreaction, reduce the release of inflammatory factors, inhibit cytokine storm in a certain extent via up-expression of IL-10 and TGF-β, more details about the mechanism of OM-MSCs lysate on ALI in this paper remain to be further clarified.

## Conclusion

Collectively, we disclosed that continuous OM-MSCs lysate administration suppressed the inflammatory response and improved symptoms in experimental ALI.

## Supplementary Information

**Additional file 1. **

## Data Availability

All data generated or analyzed during the current study are included in this article.

## Abbreviations

ALI: Acute liver injury
MSCs: Mesenchymal stem cells
OM-MSCs: Olfactory mucosa-derived mesenchymal stem cells
PBS: Phosphate buffer solution
LPS: Lipopolysaccharide
LO-2: Human normal liver cells
IL-10: Interleukin-10
TNF-α: Tumor necrosis factor-α
VEGF: Vascular endothelial growth factor
DMEM/F12: Dulbecco’s modified Eagle medium/HAM’S F12
FBS: Foetal bovine serum
TGF-β: Transforming growth factor-beta
SHH: Sonic hedgehog
SDS-PAGE: Polyacrylamide gel electrophoresis
MPO: Myeloperoxidase
ALT: Alanine aminotransferase
AST: Aspartate aminotransferase
H&E: Hematoxylin and eosin
PVDF: Polyvinylidene fluoride
HRP: Horseradish Peroxidase
ANOVA: Analysis of variance
TJP: Tight junction protein
ADSCs: Adipose tissue‑derived mesenchymal stem cells
ALI: Acute liver failure
