# Supplementary material for: Olfactory mucosa tissue-derived mesenchymal stem cells lysate ameliorates LPS-induced acute liver injury in mice
Source: BMC Pulm Med. 2022 Nov 11;22:414. doi: 10.1186/s12890-022-02204-7 (PMC9652900; doi:10.1186/s12890-022-02204-7)
Supplement: Supplementary file 1 — Additional file 1. [file 12890_2022_2204_MOESM1_ESM.docx]

Supplementary Materials for

**Olfactory mucosa tissue-derived mesenchymal stem cells lysate ameliorates LPS-induced acute liver injury in mice**

Zhe Wang2,†,, XingXing Zhang2,†, Liuyao Qi2, Wenjing Feng2, Yahan Gu2, Yuting Ding1,*

1 Department of Rehabilitation, Changshu No. 2 People’s Hospital,

Changshu Hospital affiliated the Xuzhou Medical University, Changshu, Jiangsu, China.

2 School of Medicine, Jiangsu University, Zhenjiang 212013, P. R. China

†These two authors contributed equally to this work

*Corresponding author at: Department of Rehabilitation, Changshu No. 2 People’s Hospital, No.68, Haiyunan Road, Changshu District, Suzhou, China

Email address：(Y.Ding) 373357671@qq.com

**The wold file includes:** **Fig. S1 to S3**

**Raw data of Western Blotting**

**Figure.S1**

sample order:1.Control 2.Lysate

|   35kd  **IL-10** |   35kd  **TGF-β** |  |  |
| --- | --- | --- | --- |
|   35kd  **SHH** |   45kd  140kd  **CollagenⅡ** |   100kd  **Laminin** |  |

**Additional blots in Figure.S1**

| ****  180kd-  **Laminin** |  |
| --- | --- |

**Figure.S2**

sample order:1. Control 2. LPS 3. LPS+Lysate

| ****  35kd-  **IL-10** | ****  80kd-  **MPO** |
| --- | --- |
| ****  35kd-  **TNF-α** | ****  45kd-  **Actin** |

**Additional blots in Figure.S2**

sample order: 1.Control 2.Lysate 3. LPS+Lysate

| ****  35kd-  **IL-10** | ****  35kd-  **TNF-α** |
| --- | --- |
| ****  35kd-  **TNF** |  |

**Figure.S3**

sample order: 1. Control 2.Lysate 3. LPS+Lysate

| ****  25kd-  **IL-10** | ****  35kd-  **TNF-α** |
| --- | --- |
| ****  180kd-  **TJP** | ****  45kd-  **Actin** |

**Additional blots in Figure.S3**

sample order: 1. Control 2. LPS 3. LPS+Lysate

| ****  25kd-  **TNF-α** | ****  45kd-  **TNF-α** |
| --- | --- |
